# Supplementary material for: Micronized Shell-Bioaggregates as Mechanical Reinforcement in Organic Coatings
Source: Materials (Basel). 2024 Aug 21;17(16):4134. doi: 10.3390/ma17164134 (PMC11356364; doi:10.3390/ma17164134)
Supplement: Supplementary file 1 [file materials-17-04134-s001.zip › Supplementary Materials.pdf]

Supplementary Materials:

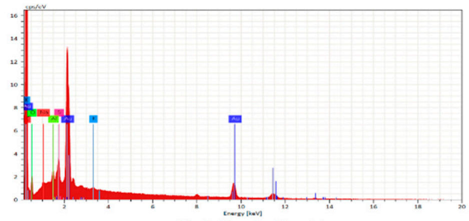

Name	Date	Time	HV [kV]	Real time [s]	Dead time [%]	Pulses [kcps]
Polytuf.spx	6/6/2024	12:56:04 PM	20.0	50.000	10	17.979

Element	At. No.	Netto	Mass [%]	Mass Norm. [%]	Atom [%]	abs. error [%] (1 sigma)	rel. error [%] (1 sigma)
Carbon	6	297280	83.82	83.82	86.75	9.02	10.76
Nitrogen	7	3030	8.97	8.97	7.96	1.57	17.55
Oxygen	8	5560	6.23	6.23	4.84	0.98	15.77
Sodium	11	1263	0.18	0.18	0.10	0.04	22.06
Aluminium	13	2611	0.21	0.21	0.10	0.04	17.53
Silicon	14	8592	0.52	0.52	0.23	0.05	9.33
Potassium	19	797	0.07	0.07	0.02	0.03	39.91
		Sum	100.00	100.00	100.00		

(**a**)

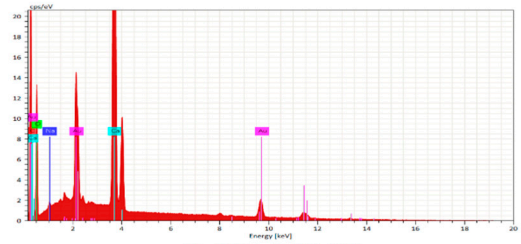

Name	Date	Time	HV [kV]	Real time [s]	Dead time [%]	Pulses [kcps]
Shells.spx	6/6/2024	1:15:45 PM	20.0	50.000	17	30.190

Element	At. No.	Netto	Mass [%]	Mass Norm. [%]	Atom [%]	abs. error [%] (1 sigma)	rel. error [%] (1 sigma)
Carbon	6	70732	17.97	27.40	42.56	2.09	11.64
Oxygen	8	36824	22.01	33.56	39.14	2.68	12.20
Sodium	11	2119	0.23	0.35	0.29	0.04	18.18
Calcium	20	455254	25.37	38.69	18.01	0.76	3.01
		Sum	65.58	100.00	100.00		

(**b**)

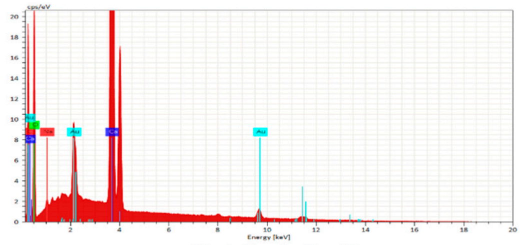

Name	Date	Time	HV [kV]	Real time [s]	Dead time [%]	Pulses [kcps]
Mussels.spx	6/6/2024	1:25:04 PM	20.0	50.000	23	41.786

Element	At. No.	Netto	Mass [%]	Mass Norm. [%]	Atom [%]	abs. error [%] (1 sigma)	rel. error [%] (1 sigma)
Carbon	6	46772	11.50	13.90	24.33	1.39	12.07
Oxygen	8	59432	31.86	38.52	50.60	3.73	11.72
Calcium	20	784500	39.09	47.27	24.79	1.16	2.98
Sodium	11	2455	0.26	0.31	0.28	0.04	17.00
		Sum	82.71	100.00	100.00		

(**c**)

Figure S1. EDXS analysis of (a) *PolyTuf® 1229*, (b) seashells and (c) mussel’s powders.
